# Evaluation of gas-sensing properties of ZnO nanostructures electrochemically doped with Au nanophases

**DOI:** 10.3762/bjnano.7.3

**Published:** 2016-01-08

**Authors:** Elena Dilonardo, Michele Penza, Marco Alvisi, Cinzia Di Franco, Francesco Palmisano, Luisa Torsi, Nicola Cioffi

**Affiliations:** 1 Department of Chemistry, Università degli Studi di Bari Aldo Moro, Bari, Italy; 2 Department of Electrotechnics and Electronics, Politecnico di Bari, Bari, Italy; 3 Italian National Agency for New Technologies, Energy and Sustainable Economic Development (ENEA), Technical Unit for Materials Technologies - Brindisi Research Center, Mesagne (BR), Italy; 4 CNR-IFN Bari, Bari, Italy

**Keywords:** Au-doped ZnO, chemiresistive gas sensor, electrosynthesis, NO_2_ gas sensor, ZnO nanostructures

## Abstract

A one-step electrochemical method based on sacrificial anode electrolysis (SAE) was used to deposit stabilized gold nanoparticles (Au NPs) directly on the surface of nanostructured ZnO powders, previously synthesized through a sol–gel process. The effect of thermal annealing temperatures (300 and 550 °C) on chemical, morphological, and structural properties of pristine and Au-doped ZnO nancomposites (Au@ZnO) was investigated. Transmission and scanning electron microscopy (TEM and SEM), as well as X-ray photoelectron spectroscopy (XPS), revealed the successful deposition of nanoscale gold on the surface of spherical and rod-like ZnO nanostructures, obtained after annealing at 300 and 550 °C, respectively. The pristine ZnO and Au@ZnO nanocomposites are proposed as active layer in chemiresistive gas sensors for low-cost processing. Gas-sensing measurements towards NO_2_ were collected at 300 °C, evaluating not only the Au-doping effect, but also the influence of the different ZnO nanostructures on the gas-sensing properties.

## Introduction

Today the use of low-cost portable gas sensors is essential to detect and to monitor toxic, polluting and combustible gases for the environmental protection. In this context, chemical gas sensors have a deep impact on human security, medical prevention and diagnosis, monitoring and detection of polluting and toxic substances [1]. Specifically, nowadays metal oxide semiconductors (MOS), such as WO_3_, SnO_2_, In_2_O_3_ and TiO_2_ [2], have been largely used as active layer in low-cost chemiresistive gas sensors, due to their high sensitivity to gaseous analytes and easy production.

The gas-sensing mechanism of MOS-based gas sensors is based on receptor and transducer functions [3]. Specifically, the first regards the recognition of a gaseous analyte by an electric charge transfer at the gas–solid interface on the MOS surface. It is influenced by the surface area and by the presence of structural defects and impurities that positively affect the gas detection. Moreover, it is favored by the presence of oxygen species adsorbed on MOS surface, whose amount strongly depends on MOS morphology and structure, and on the gaseous analyte [4].

The second function transduces the solid–gas interaction into the electrical resistance variation of the gas sensor, correlated to the adsorbed gas concentration to be detected; it is particularly influenced by the morphological structures of the MOs active layer and by the interface between sensing material and metal electrodes of the device [5]. In most cases, the surface conductance of MOS varies when exposed to oxidizing/reducing gases, usually exhibiting n-type behavior [6]. Therefore, the resistance increases in presence of an oxidizing gaseous molecules, and decreases in presence of a reducing gas.

MOS-based gas-sensing performance is positively influenced by the exposed surface. Therefore, the development of new processes to synthesize nanomaterials has improved the performance of these materials [7–9]. Moreover, it has been demonstrated that a careful control of the MOS nanostructures, used as active layers in gas sensors, permits to improve their gas-sensing properties [10–11]. Specifically, the smaller grain size influences the material resistivity, so that the conductive properties of the material strongly depend on the character of the surface [12–13].

Generally, MOS sensing layers are thermally stabilized over 300 °C, although high temperatures cause grain growth, which negatively affects the surface to volume ratio of the sensing layer [14]. However, high crystalline nanostructures are less affected by this drawback, improving the stability of gas sensors [9,15–16]. Additionally, also the use of one-dimensional nanostructures (e.g., nanorods, nanowires, and nanobelts), with high surface to volume ratio, improves the gas-sensing properties [9,17].

Among different MOS, ZnO has been widely used as a gas-sensing material because of its remarkable properties, such as high chemical and thermal stability, wide direct band gap, chemical sensitivity to different adsorbed gases, highly mobile conduction carriers, non-toxicity and low cost [18–20]. Moreover, since gas-sensing mechanism is a surface-related phenomenon, nanosized ZnO is now largely used as gas-sensing material thanks to its high surface area. Up to now, different strategies have been proposed to produce ZnO based on various forms, including thin-films, nanoparticles, and one-dimensional (1D) nanomaterials [21–23].

MOS in general and ZnO nanomaterials in particular are promising as sensing layer in chemiresistive gas sensors, although their limited selectivity, high response/recovery time, high-power consumption, and lack of long-term stability have limited their use in more demanding applications [24]. Nowadays, many strategies have been developed to improve the gas-sensing properties of MOS-based gas sensors. These include the synthesis of porous nanoparticles [25–26] the assembly of hierarchical structures [27–28], the use of catalysts and promoters [29–30], multi-sensor array systems [31], the optimization of the operating temperature of the sensors [32], cycled temperature operation, and the use of nanotechnology. Among these, the use of dopants and/or catalytic elements has been considered an effective way to improve the sensor performance [33]. Specifically, the loading of MOS with noble metals (e.g., Au, Pt, and Pd) that act as sensitizers or promoters, is an effective method to catalyze the gas-sensing reactions [34]. Until now, many strategies have been developed to deposit noble metals onto MOS matrices [35–40], and, although most of them are unique and effective, they are also complex and time-consuming; furthermore, metal nanoparticles prepared in these way can undergo undesired clustering, thus lowering their catalytic activity [41].

In this contribution, sol–gel pre-synthesized and dried ZnO powder was directly functionalized with Au NPs of controlled size and loading by means of an in situ electrodecoration procedure based on the so called sacrificial anode electrolysis (SAE) [42–43]. Subsequently, ZnO and Au@ZnO nanocomposites, annealed at two different temperatures, 300 and 550 °C, were morphologically and chemically characterized by means transmission and scanning electron microscopy (TEM, SEM), as well as X-ray photon electron spectroscopy (XPS), revealing the successful decoration of ZnO spherical and rod-like nanostructures, obtained at 300 and 550 °C, respectively, with nano-phase gold at the elemental oxidation state.

The main focus of this study is to investigate the influence of ZnO morphology and of Au-doping on the gas-sensing capabilities, taking into the account the importance of the annealing temperature in defining the morphology and the chemical composition. It was found that the NO_2_ responses of un-doped and Au-doped spherical-like ZnO nanostructures were lower than those of pristine and Au-doped ZnO nanorods, revealing that for NO_2_ gas-sensing the rod-like structure and the intimate contact between stabilized Au NPs and ZnO nanorods have a significant positive effect on the resistance of sensors and, consequently, their response.

## Experimental

### Sol–gel synthesis of ZnO

ZnO nanostructures were prepared through a sol–gel process. An aqueous solution of ZnCl_2_ (0.1 M) was heated to 60 °C for 1 h in a water-bath under continuous stirring. Ammonia solution (0.1 M) was then added drop-wise until a dense gel was formed at pH 9 [43]. The gel was washed with distilled water until complete elimination of chlorine ions in the liquid phase and then dehydrated at 120 °C for 2 h to maintain hydroxy (–OH) groups on the oxide surface, that are ideal to stabilize Au nanoparticles during the electrosynthesis [43–46].

### Electrochemical decoration of ZnO by Au NPs

Au@ZnO hybrid nanostructures were prepared by SAE procedure carried out under inert (N_2_) atmosphere, using a three-electrode cell equipped with an Au anode, a Pt cathode, and an Ag/AgNO_3_ (0.1 M in acetonitrile) reference electrode [43,47]. The electrodes with area of about 1.25 cm^2^ were immersed in the electrolyte solution (0.05 M in 5 mL) of vacuum dried tetraoctylammonium chloride (TOAC), which acts both as electrolyte and Au NPs stabilizer, in anhydrous tetrahydrofuran (THF) and acetonitrile (ACN) mixed in 3:1 ratio. The dried ZnO powder (about 1 g) was added as support particles into the electrolytic cell and stirred to yield a homogeneous ZnO suspension. The electrolysis was performed in potentiostatic mode, fixing the potential of the working electrode at 1 V, and stopped after the total charge reached 300 C [48]. Subsequently, the Au@ZnO nanocomposite was centrifuged at 6000 rpm to separate unsupported colloidal Au nanoparticles from the heavier Au@ZnO nanocomposite. After separation, Au@ZnO were thermally annealed at 300 or 550 °C for 2 h in air to study the effect of thermal annealing on the structural stability of Au NPs [45], and of ZnO [44,49], and on the surface chemical composition of electro-decorated ZnO nanostructures.

### Material characterization

A Thermo VG Theta Probe XPS spectrometer (μ-spot monochromatic Al Kα source) was used for surface chemical analysis. The XPS survey spectra were acquired in fixed analyzer transmission mode with 150 eV pass energy, and the XPS high-resolution spectra with 100 eV pass energy. The reproducibility was evaluated by replicating the analysis five times in different points on selected samples.

The morphological analyses of pristine and Au-doped ZnO nanomaterials were performed by a FEI TECNAI T12 TEM instrument operated at 120 kV and by a field emission Zeiss ΣIGMA SEM operated at 5–10 kV, 10 μm aperture.

### Chemiresistive sensor preparation and gas-sensing set-up

**Figure 1:**
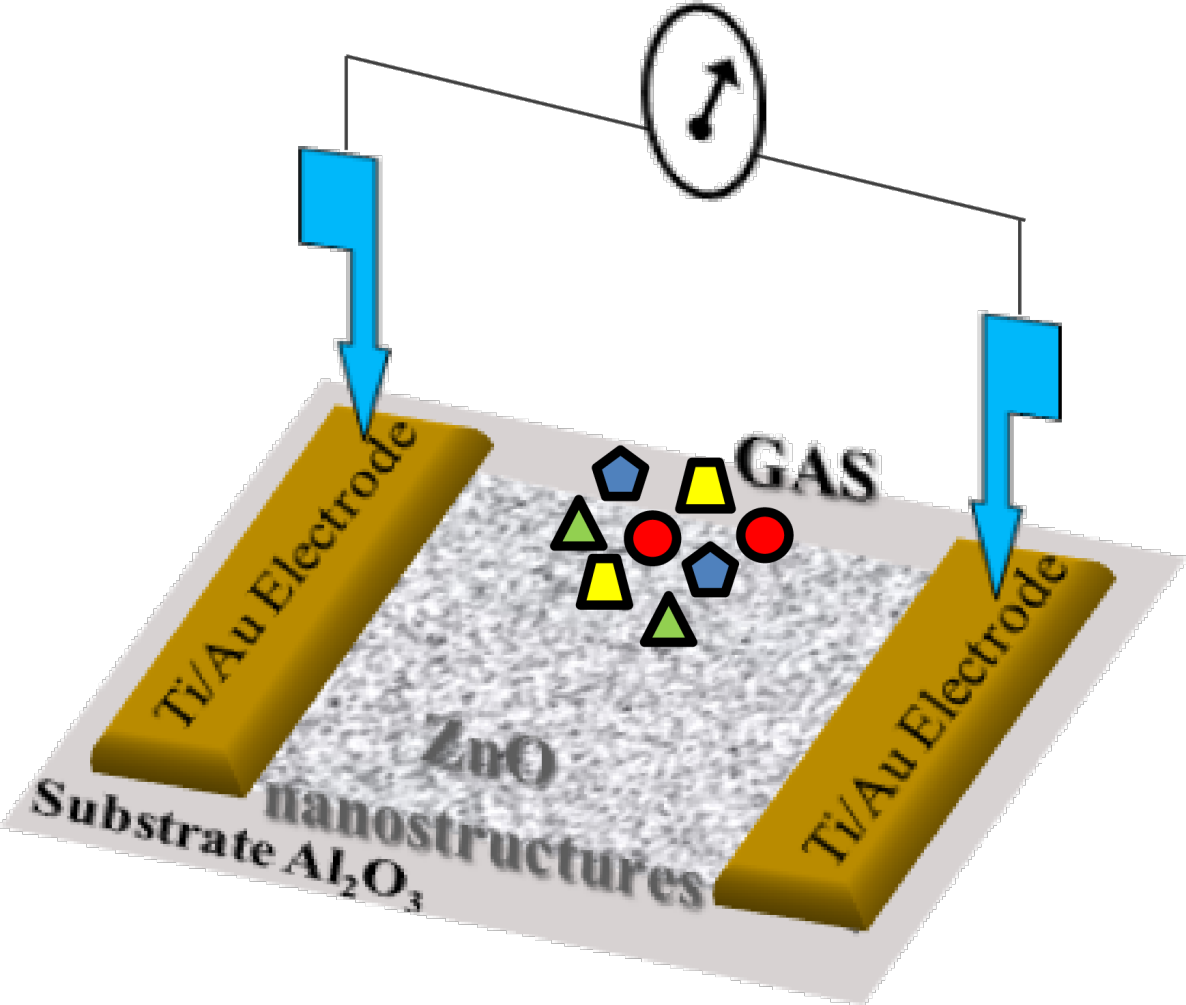
A scheme of ZnO-based chemiresistive gas sensor.

A scheme of the used chemiresistive ZnO-based gas sensor is shown in Figure 1.

After the thermal annealing at 300 or 550 °C for 2 h, pristine and Au-doped ZnO, were redispersed in ACN solution and subsequently drop-casted as sensing layers between the Au contacts of the sensor device. Finally, the device was heated at 300 °C for 2 h, to provide an additional stabilization to the sensor active material. The experimental set up used for gas-sensing measurements is reported elsewhere [50]. Dry air was used as the reference gas and to dilute the targeted gas, keeping the total flow rate constant at 1000 sccm. Distinct mass flowmeters (MFCs) at different full scales and controlled by a software were used. The gas-sensing experiments were performed by measuring the resistance change of the sensing layer during the exposure to the targeted gas, at an operating temperature of 300 °C. The gas-sensing cycle consisted of a period of 60 min to stabilize the sensor signals under dry air flow, an exposure time of 10 min to various targeted gas concentrations at decreasing steps and finally a recovery time of 30 min to restore the sensor signal to the initial value under dry air flow and to clean the test cell and sensor surface. The sensor response to a given gas concentration is defined as the relative resistance change, Δ*R/R_i_* (%), where Δ*R* is the change in resistance between the values of steady-state of the electrical resistance of the sensor upon a target gas and in air, *R_f_* and *R_i_*, respectively. The mean gas sensitivity, *S*_m_ (% ppm^−1^), is defined as weighted mean of the ratio between percentage relative resistance change (%) over gas concentration unit (ppm); it can be calculated by Equation 1:

[1]
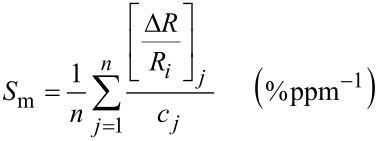


where *c_j_* is a defined gas concentration to which [Δ*R/R_i_*]*_j_* is the corresponding response. The response time is defined as the time necessary to reach the 90% of the resistance variation under the presence of the gaseous analyte with respect to the initial equilibrium resistance value. The recovery time is defined as the time necessary to reach the 90% of the original resistance value in air without the gaseous analyte.

## Results and Discussion

### Chemical and structural properties

**Figure 2:**
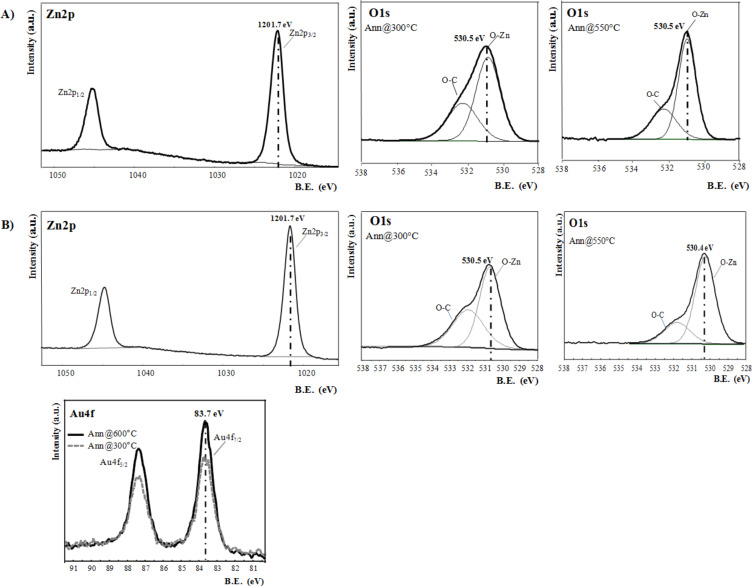
A) XPS spectra of the chemical elements in pristine ZnO: Zn 2p spectrum and O 1s spectra, deconvoluted in two components (O–Zn and O–C), of ZnO annealed at 300 and 550 °C. B) XPS spectra of the chemical elements in hybrid Au@ZnO: Zn 2p spectrum, and Au 4f and O 1s spectra, the last deconvoluted in the two components, after annealing at 300 and 550 °C.

The surface chemical composition of pristine and Au-doped ZnO nanocomposites annealed at 300 and at 550 °C was obtained by XPS analysis. In Figure 2A the high resolution XPS spectra of Zn 2p and O 1s in pristine ZnO are reported, evaluating particularly the variation of the components of O 1s signal, the oxygen linked to the metal (O–Zn at 530.5 eV) and the oxygen linked to carbon (O–C at 532.5 eV), at the two annealing temperatures. In Figure 2B the high resolution XPS spectra of the, Zn 2p, O 1s and Au 4f in Au@ZnO hybrid structures are reported, investigating, also in this case, how the annealing temperature affects the O 1s spectrum and its components, and on the Au 4f spectrum.

In all cases, for pristine and Au-doped ZnO, the Zn 2p spectrum is unchanged after annealing at 300 and 500 °C; instead, the relative area of the two components of O 1s spectrum, O–Zn and O–C, are changed at the two different annealing temperature: The content of O–Zn component increases respect that of O–C at higher annealing temperature. This trend is explainable by the burning of carbonaceous species at high temperature, also demonstrated by the reduction of carbon content in the Table 1 reported below.

**Table 1:** XPS surface chemical composition of pristine and Au-functionalized ZnO, annealed at 300 and 550 °C. The O–M percentage refers to the atomic percentage of oxygen bound to metal.

	ZnO Ann@300 °C	Au@ZnO Ann@300 °C	ZnO Ann@550 °C	Au@ZnO Ann@550 °C

C%	19.2 ± 0.5	15.4 ± 0.5	16.2 ± 0.5	12.0 ± 0.5
O_(total)_%	49.1 ± 0.5	43.0 ± 0.5	44.2 ± 0.5	46.0 ± 0.5
O–M%	31.5 ± 0.5	37.0 ± 0.5	39.5 ± 0.5	40.1 ± 0.5
Zn%	31.7 ± 0.5	38.5 ± 0.5	39.6 ± 0.5	40.5 ± 0.5
Au%	–	1.3 ± 0.2	–	1.5 ± 0.2
Cl%	–	1.0 ± 0.5	–	–
N%	–	0.8 ± 0.5	–	–

In hybrid systems, the binding energy of the Au 4f_7/2_ peak was 83.7 ± 0.2 eV, which is lower than that of bulk metallic Au at 84.0 eV, independent of the annealing temperature. This is a well-known effect attributed to initial state size-effects in Au NPs of very small dimension [43]. The detailed elemental composition of ZnO, prepared through sol–gel method and annealed at 300 or 550 °C, is reported in Table 1. After the annealing at the two different temperatures, in both cases, the O–Zn/Zn atomic ratio (the percentage of oxygen bound to metal divided by the total metal percentage) was equal to 1 (stoichiometric) at both annealing temperatures. Table 1 reports for Au@ZnO nanocomposites annealed at 300 °C a low atomic percentage of N 1s and Cl 2p elements, originating from the TOAC stabilizer of Au NPs, that still remained on the surface of Au NPs. The presence of these elements was not revealed in Au@ZnO nanocomposites annealed at 550 °C, because the traces of TOAC were completely removed after the annealing at higher temperature. Moreover, after the annealing the carbon percentage decreased, confirming that all the surfactant shell was partially (at 300 °C) or almost completely (at 550 °C) removed trough thermal annealing. The total amount of elemental gold (Au^0^) deposited on ZnO by SAE process was about 1.2–1.5 atom %.

**Figure 3:**
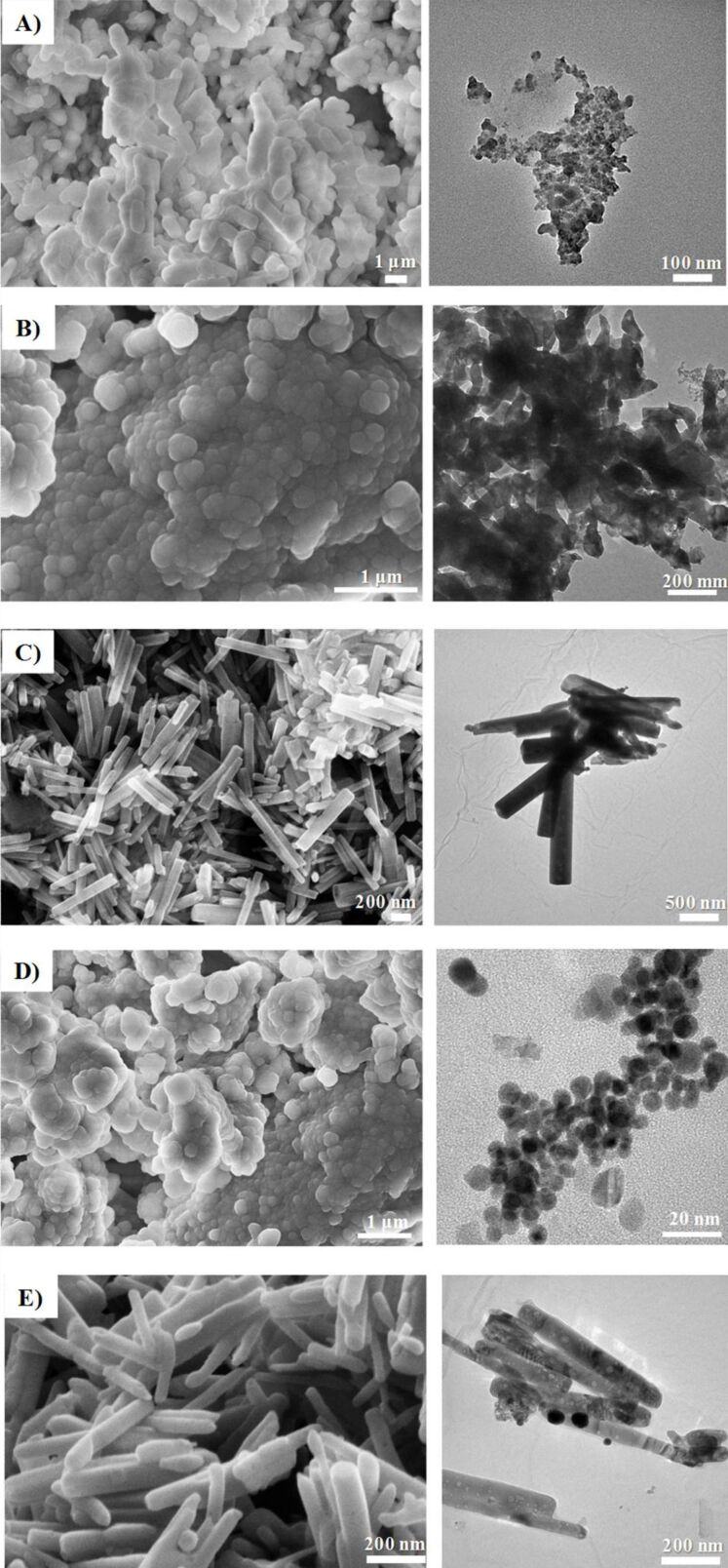
SEM (left side) and TEM (right side) images of ZnO A) dried at 120 °C, and annealed at B) 300 °C and C) 550 °C, and of Au@ZnO hybrid systems annealed at D) 300 °C and E) 550 °C.

In Figure 3A–C the morphological evolution of ZnO structures, dried at 120 °C and annealed at 300 and 550 °C is reported. In particular, the dried ZnO powder, used in the electrochemical decoration process, revealed an amorphous morphology. Furthermore, at low annealing temperature (300 °C) the structure of ZnO became spherical, while, at 550 °C, ZnO was stabilized in rod-like structures. In Figure 3D,E the SEM and TEM images of Au@ZnO annealed at 300 and 550 °C, respectively, are reported. In both cases ZnO nanostructures were successfully decorated with spherical Au NPs. In the case of Au@ZnO annealed at 300 °C, the density of Au NPs on ZnO nanostructures was higher than that in the case of hybrid nanocomposites annealed at 550 °C in which the surface of ZnO nanorods was decorated with only few Au NPs.

The annealing temperature strongly affects the ZnO morphology and crystallinity, the distribution of Au dopants on the ZnO nanostructures, and the chemical composition at the interface between the two systems; therefore, it should strongly influence the ZnO properties concerning the gas adsorption and reactivity, as discussed in the next paragraph.

### Gas-sensing properties

**Figure 4:**
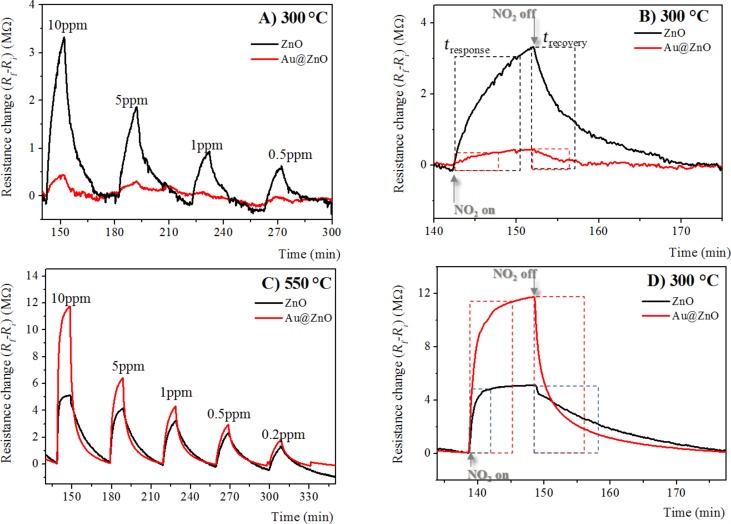
A) Time response of chemiresistors based on pristine and Au-doped ZnO annealed at 300 °C, exposured to different NO_2_ concentrations [0.5–10 ppm] at operating temperature of 300 °C; B) enlarged parts of the dynamic response curve shown in a at a NO_2_ concentration of 10 ppm drawn to reveal the moments of gas input and gas stop. C) time response of chemiresistors based on pristine and Au-doped ZnO annealed at 550 °C, exposed to different NO_2_ concentrations [0.2–10 ppm] at an operating temperature of 300 °C; D) enlarged parts of the dynamic response curve shown in A at a NO_2_ concentration of 10 ppm drawn to illustrate the moments of gas input and gas stop.

In Figure 4A and Figure 4B, the time responses of the electrical resistance change of gas sensors based on pristine and Au-doped ZnO nanostructures annealed at 300 and 550 °C, respectively, are reported at different NO_2_ concentrations [0.5–10 ppm] and at an operating temperature of 300 °C.

All films exhibit n-type behavior: the resistance increases in presence of an oxidizing gas such as NO_2_. Moreover, as expected, the sensor responses increase upon increasing NO_2_ gas concentration. The resistance increased upon exposure to NO_2_ gas, and recovered completely to the initial value after the removal of NO_2_ gas. Good reproducibility and stability of NO_2_ gas sensor responses were revealed for repeated test cycles. Figure 4B and Figure 4D, respectively, show an enlarged part of the data in panels A and C measured at 10 ppm NO_2_ for pristine and Au-doped ZnO annealed at 300 and 550 °C to illustrate the moments of gas input and gas stop.

As written in the Introduction, the gas-sensing mechanism of ZnO involves chemical and electronic interaction between the gas and the ZnO at the oxide surface, revealed as the resistance variation of the sensing materials. Charge transfer between oxygen molecules absorbed on the ZnO surface forms O_2_^−^, O^−^ and O^2−^ ions. When NO_2_ molecules are absorbed, a negatively charged NO_2_^−^ species is formed at the ZnO surface, resulting in the increase of the resistance of ZnO. Subsequently, NO_2_ gas is desorbed after the reaction of NO_2_^−^ with absorbed oxygen, and O^2−^ ions are adsorbed. Therefore, after the total desorption of the NO_2_ gas, the ZnO resistance decreases down to its original value.

**Figure 5:**
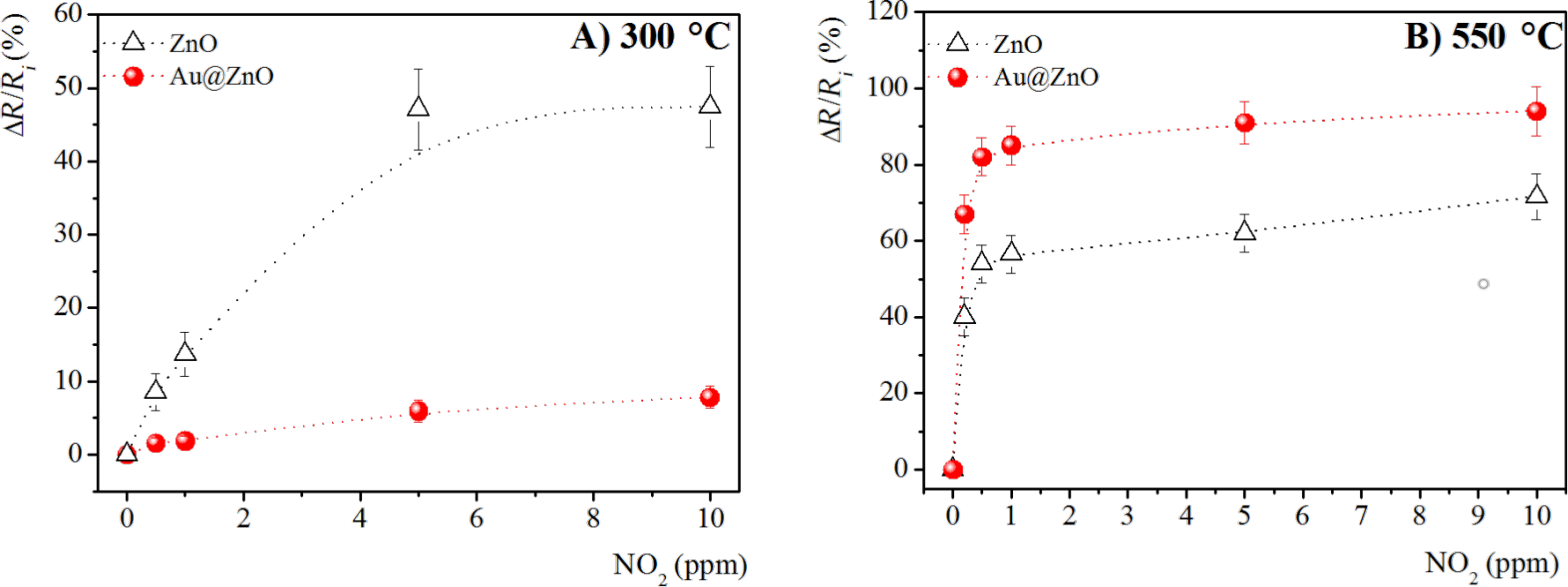
Calibration curves in terms of the percentage relative electrical resistance change for a chemiresistor based on pristine and Au@ZnO annealed at A) 300 °C and B) 550 °C, towards NO_2_ gases, at a working temperature of 300 °C.

As reported in the calibration curves in Figure 5, the sensing response of pristine and Au-doped ZnO nanostructures are strongly influenced by the annealing temperatures. For samples annealed at 300 °C, the presence of Au NPs did not improve the gas sensing properties, instead it reduced the sensitivity of ZnO. In contrast, the ZnO sensitivity was improved in Au-doped nanocomposites annealed at 550 °C, allowing the NO_2_ detection down to a concentration of 0.2 ppm.

Comparing the pristine ZnO nanostructures annealed at the two different temperatures the responses of ZnO nanorods formed at 550 °C were higher than those of ZnO nanospheres obtained at 300 °C. Thus, the poor response of ZnO nanospheres compared to ZnO nanorods, although their surface area is higher, is possibly related to transducer function. Rai et al. [5] reported that the presence of a great number of grain boundaries in ZnO nanospheres acts as a highly resistive barrier, inducing the increase of the overall device resistance, since the electrical conductivity is equally influenced by the density and mobility of the charge carriers in the material. Moreover, the response times of pristine ZnO nanorods at different NO_2_ concentrations are lower compared to that of ZnO nanospheres, although their recovery times are higher, as reported in Table 2.

**Table 2:** Comparison of the response time (*t*_response_) and recovery time (*t*_recovery_) between spherical and rod-like pristine and Au-doped ZnO at various NO_2_ concentrations.

NO_2_ (ppm)	ZnO 300 °C	Au@ZnO 300 °C	ZnO 550 °C	Au@ZnO 550 °C
*t*_response_ (s)				

10	438 ± 30	336 ± 30	132 ± 25	240 ± 20
5	444 ± 30	474 ± 30	240 ± 25	300 ± 20
1	414 ± 30	486 ± 30	360 ± 30	420 ± 20
0.5	336 ± 30	450 ± 30	420 ± 30	450 ± 20

*t*_recovery_ (s)				

10	792 ± 30	342 ± 30	1470 ± 30	654 ± 20
5	984 ± 30	754 ± 30	1320 ± 30	840 ± 20
1	540 ± 30	630 ± 30	1140 ± 30	936 ± 20
0.5	504 ± 30	587 ± 30	900 ± 30	1020 ± 20

**Figure 6:**
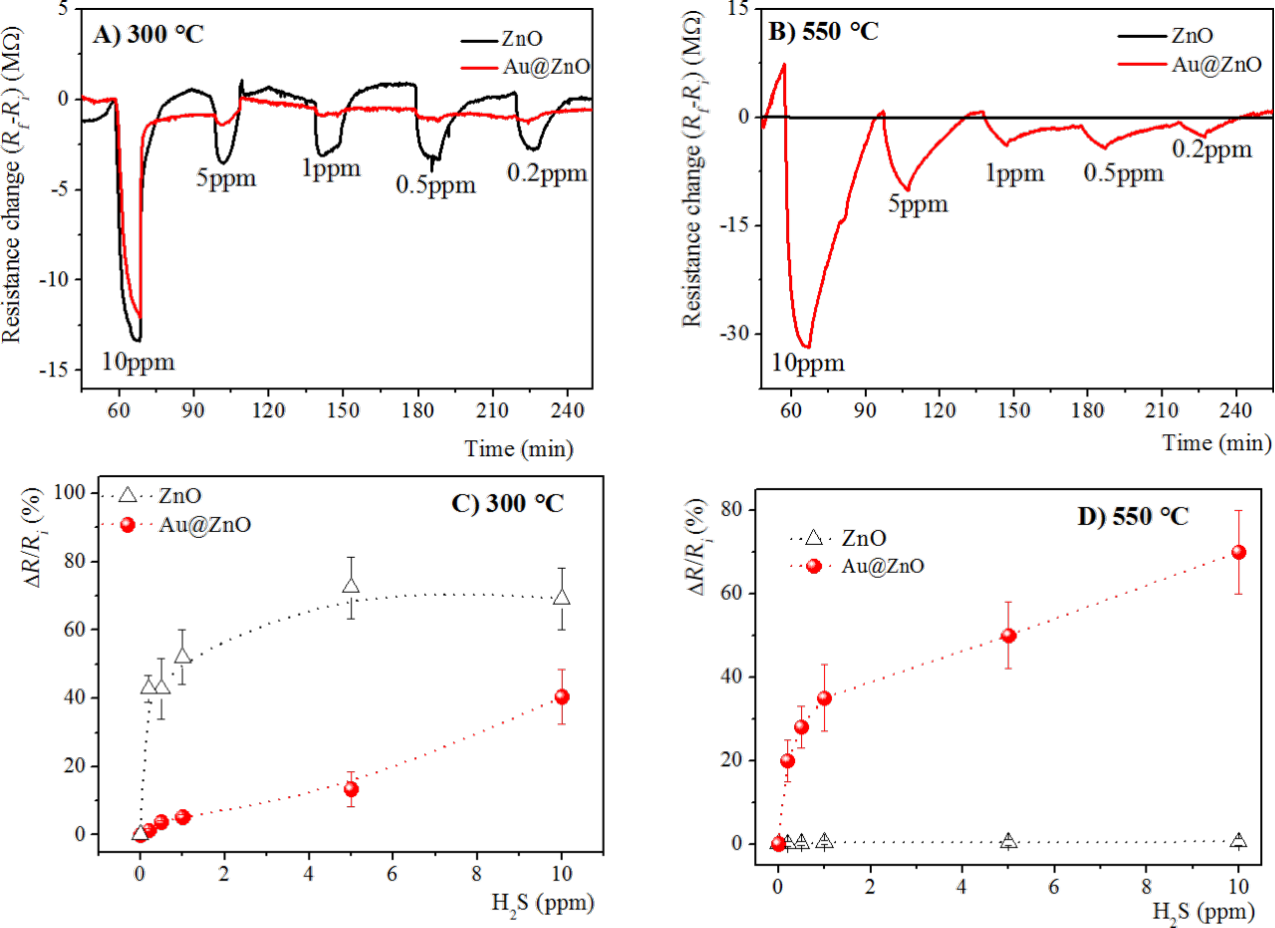
Gas sensor resistance of pristine and Au@ ZnO annealed at A) 300 °C and B) 550 °C over time under exposure to different H_2_S concentrations [0.2–10 ppm] at operating temperature of 300 °C. Calibration curves of gas sensors, based on of pristine and Au@ ZnO annealed at C) 300 °C and D) 550 °C, to H_2_S gas [0.2–10 ppm] at an operating temperature of 300 °C.

H_2_S detection by means of ZnO nanostructures was investigated, as well. In contrast to the previous case, ZnO nanospheres provided a better response to the H_2_S analyte and this is probably because of the higher potential barrier than that of the ZnO nanorods. The time responses of gas sensors based on pristine and Au-doped ZnO nanostructures annealed at 300 and 550 °C are reported in Figure 6A and Figure 6B, respectively, at different H_2_S concentrations (0.2–10 ppm), at an operating temperature of 300 °C. The response decreases with decreasing gas concentrations in both devices.

As reported in the calibration curves in Figure 6C and Figure 6D, the sensing response of pristine and Au-doped ZnO nanostructures were strongly influenced by the annealing temperature. Similarly to the trend reported for NO_2_ detection, for samples annealed at 300 °C, the presence of Au NPs did not improve the gas-sensing properties, instead the sensitivity of ZnO decreased. In contrast, for samples annealed at 550 °C, only the presence of Au NPs on the surface of ZnO nanorods allowed for the detection of H_2_S down to a concentration of 0.2 ppm. Finally, the response intensity towards H_2_S gas provided by ZnO nanospheres, formed at 300 °C, were higher than those of ZnO nanorods, obtained at 550 °C. This is because for ZnO nanospheres *R_i_* in air is high, therefore in presence of a reducing gas, such as H_2_S, the negative variation of the resistance (*∆R*) is greater, hence its response is also higher.

**Figure 7:**
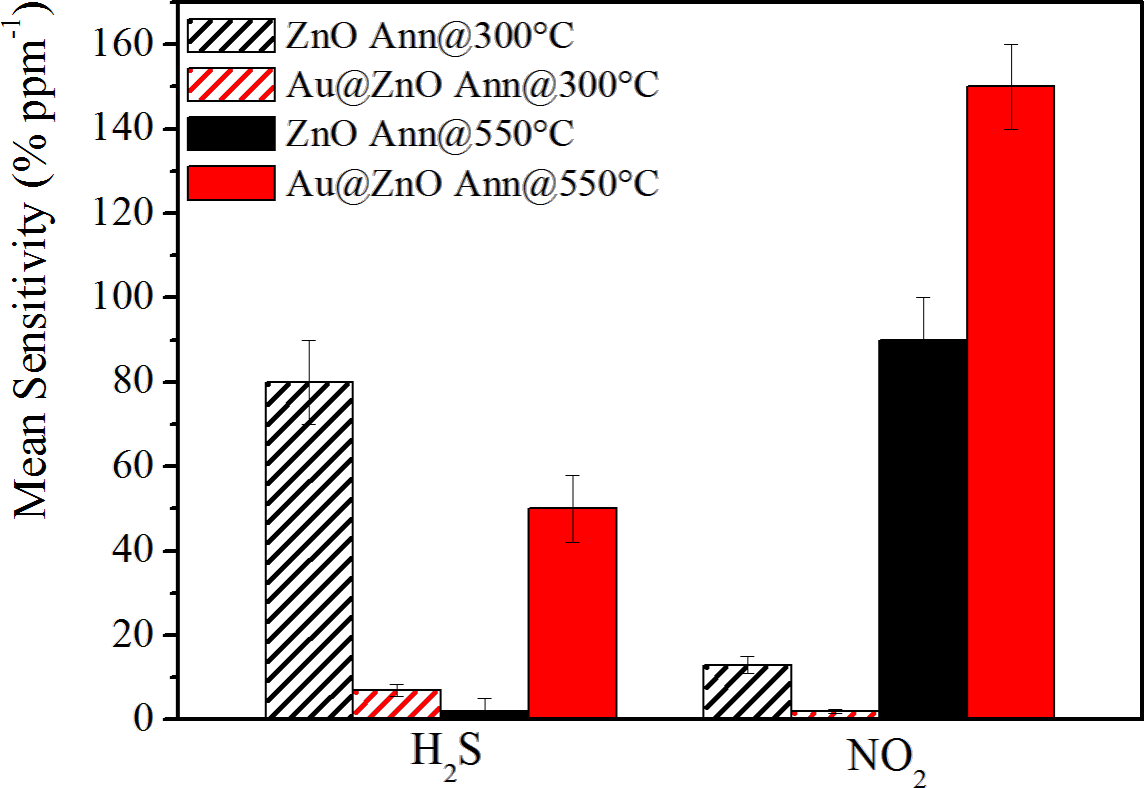
Mean sensitivity of pristine and Au@ZnO annealed at 300 and 550 °C towards H_2_S and NO_2_ gases at an operating temperature of 300 °C.

In Figure 7 the mean sensitivity of pristine and Au@ZnO annealed at 300 and 550 °C towards H_2_S and NO_2_ gases at an operating temperature of 300 °C is reported.

The mean sensitivity of Au-doped ZnO nanorods obtained at 550 °C is always higher than that of pristine ZnO for both analyzed gases, therefore the catalytic effect of Au NPs positively affects both NO_2_ and H_2_S gas sensing. In contrast, considering the pristine and Au-functionalized ZnO nanostructures annealed at 300 °C, the un-functionalized ZnO nanospheres have a sensitivity higher than the Au-functionalized ones. Moreover, ZnO nanorods, both non-functionalized and Au-functionalized, are selective to NO_2_ gas monitoring at an operating temperature of 300 °C, while pristine ZnO nanospheres, annealed at 300 °C, are selective to H_2_S gas monitoring at a sensor temperature of 300 °C.

The following two conclusions can be extracted from the above results: (1) rod-like ZnO is more favorable than spherical ZnO for NO_2_ gas sensing; and (2) the annealing temperatures influence not only the morphology but also the surface chemistry, especially of the Au-functionalized nanostructures, having a larger effect, as a consequence, on the sensing response of the nanostructures.

## Conclusion

This manuscript reports the successful electrochemical surface decoration of ZnO nanostructures by Au NPs, which were subsequently thermally annealed at 300 and 550 °C. Pristine and Au@ZnO nanostructures were chemically and morphologically characterized. The gas-sensing measurements of ZnO and Au@ZnO used as sensitive layers in chemiresistive gas sensors, revealed that rod-like ZnO nanostructures obtained at 550 °C are selective in NO_2_ gas monitoring at an operating temperature of 300 °C. In addition, the Au-doping improved the sensitivity only in rod-like ZnO nanostructures obtained at 550 °C. On the contrary, in ZnO nanostructures annealed at 300 °C, the spherical structures and the residual presence of electrolyte on Au NPs surfaces influence the gas-sensing response, yielding the worst sensor response towards NO_2_.

Future work will be addressed to electrochemically functionalize ZnO nanocomposites with other noble metals, such as Pd, to improve the sensor sensitivity and selectivity towards other toxic and pollutant gases, such as hydrocarbons.
